# Adsorption and Diffusion of Cisplatin Molecules in Nanoporous Materials: A Molecular Dynamics Study

**DOI:** 10.3390/biom9050204

**Published:** 2019-05-27

**Authors:** Marjan A. Nejad, Herbert M. Urbassek

**Affiliations:** Physics Department and Research Center OPTIMAS, University Kaiserslautern, Erwin-Schrödinger-Straße, D-67663 Kaiserslautern, Germany; ahmadi@rhrk.uni-kl.de

**Keywords:** molecular dynamics, nanopores, cisplatin, targeted drug-delivery systems

## Abstract

Using molecular dynamics simulations, the adsorption and diffusion of cisplatin drug molecules in nanopores is investigated for several inorganic materials. Three different materials are studied with widely-varying properties: metallic gold, covalent silicon, and silica. We found a strong influence of both the van der Waals and the electrostatic interaction on the adsorption behavior on the pore walls, which in turn influence the diffusion coefficients. While van der Waals forces generally lead to a reduction of the diffusion coefficient, the fluctuations in the electrostatic energy induced by orientation changes of the cisplatin molecule were found to help desorb the molecule from the wall.

## 1. Introduction

Among the platinum-based anticancer drugs, cisplatin (cis-diamminedichloroplatinum(II), Pt(NH${}_{3}$)${}_{2}$Cl${}_{2}$) plays an important role [1] and has been used since the 1970s to treat a wide array of cancers [2,3,4,5], despite several severe side effects [6]. Targeted drug-delivery systems may provide a promising route for minimizing the harmful side effects by using carrier systems (nanocontainers), which transport the drug to the tumor cells that are the targets of the treatment [7]. The choice of an appropriate container material is of obvious relevance for this method; besides biocompatibility, the controlled release of the drug from the container must be guaranteed [8].

While nanoporous materials are obvious candidates for nanocontainers, for a controlled drug release, the diffusion of the drug in the nanopores, as well as its adhesion to the walls must be studied. Here, atomistic simulations, in particular, those based on molecular dynamics (MD), may assist our understanding, since the interatomic interactions governing the drug behavior are well reproduced in available force fields. Thus, previous MD studies investigated water and drug diffusion, as well as drug-wall interactions in silica nanopores [9] and in carbon nanotubes [10,11]

Various inorganic materials are used in medicine for drug-delivery systems, among them silver, gold, iron oxide, silica, and silicon [12]. Among this variety, we have chosen gold because it features good biofunctionalization properties; gold nanoparticles can be functionalized with DNA molecules, peptides, and antibodies via covalent or non-covalent interactions, thus rendering gold a powerful option for drug-delivery carriers [13,14]. In addition, gold nanoparticles can also be easily conjugated to antibodies and are used as an effective agent for diagnostics and therapy [15]. Besides metallic Au, we also chose a purely covalently bonded material, silicon. Furthermore, silicon nanoparticles are good candidates for drug-delivery systems because of their high biocompatibility, biodegradability, and high surface area [16]. Due to the optical properties of silicon, they are also employed for in vivo imaging without the need for labeling [17]. For example, luminescent silicon quantum dots are being used for bio-imaging [18]; when coated with dye material, the enhanced photo-luminescence of the dye allows tracking the path of the drug molecule through the body [19]. Finally, we also studied silica nanopores as this material possesses a high biocompatibility [20,21,22]. Several previous studies demonstrated the applicability of mesoporous silica nanoparticles, including functionalized variants, for targeted drug delivery [8,23,24].

In this study, we used atomistic modeling to investigate the diffusivity of cisplatin in three different inorganic nanopores. We chose materials from very different material classes: gold, silica, and silicon. To characterize the influence of wall adsorption during the diffusion process, we calculated the interaction energy between the cisplatin molecule and the pore wall.

## 2. Methods

Three different inorganic materials were studied: Au, silicon, and silica; the results of amorphous silica can be compared to crystalline silica in the form of cristobalite [25]. The force fields of these materials were selected such that the interaction with water molecules was reproduced reliably. For silica, we hence chose the force field provided by [26]. The interaction with water is described by a van der Waals (vdW) interaction with a well depth of 0.3 kcal/mol superposed on an electrostatic interaction with Si and O charges chosen as +1e and −0.5e, respectively; *e* is the elementary charge. We also used a pure Si crystallite; its interaction was adopted from the same reference [26]. It interacts with water by a purely vdW interaction with a well depth of, again, 0.3 kcal/mol. For Au, the force field parameters developed by Heinz et al. [27] were adopted. The interaction with water is here a pure vdW interaction with a large well depth of 5.3 kcal/mol; this value has been determined by fitting to the experimentally-determined Au-water interface tension. This potential was implemented in the CHARMM force field and allowed modeling the interface of Au with both organic molecules and inorganic components.

The materials were created in the form of blocks with a length of 83 Å and lateral sizes of 40 Å × 40 Å. The surfaces of the Si and Au crystals have (001) orientations, in order to compare with our recent study of cristobalite [25]. The amorphous silica block was provided by the “Inorganic Builder” VMD plug-in [28]. In the middle of the 40 Å × 40 Å front face of the block, we drilled a cylindrical hole with a radius of R=10 Å and a length of 83 Å. In the case of crystalline SiO${}_{2}$, we chose the crystal structure of α-cristobalite; we studied both a pore running perpendicular to the polar (100) surface (termed “axial” pore) and to the non-polar (010) surface surface (termed “transverse”). We made sure that the remaining silica specimen was electrically neutral; however, it carried an electrical dipole moment. In the case of a-SiO${}_{2}$, it amounted to 3496 Debye and was directed at an oblique angle to the pore axis; for the axial pore, it ran along the pore axis (10,259 Debye) and for the transverse pore, perpendicular to it (13,836 Debye).

The porous block was embedded in a pure water environment of (modified) TIP3P [29,30] water molecules. The simulation volume had a thickness of 120 Å and a lateral extension of 50 Å × 50 Å, such that the block was entirely flooded with water. We used periodic boundary conditions to get rid of any boundary effects. We made sure that the water penetrated homogeneously inside the cylindrical holes. The entire system was relaxed for 20 ps for equilibration.

A cisplatin molecule, Pt(NH${}_{3}$)${}_{2}$Cl${}_{2}$, was initially put in the middle of the pore with random orientation. Note that this situation may appear special, since the molecule would later also adsorb at the pore walls; however, our trajectories showed that the molecule sufficiently performed many phases of alternating adsorption and free motion, such that this special initial condition did not strongly bias our results. The cisplatin force field was taken as described recently [25]. Cisplatin interacted via electrostatic and vdW forces with the water molecules and the wall surfaces; the Lennard–Jones parameters describing the vdW interaction were obtained from the usual Lorentz–Berthelot mixing rules. The cisplatin molecule carries partial charges at Pt (+0.71), N (−0.64), Cl (−0.55), and H (+0.28). The vdW size of the cisplatin molecule amounted to 5.8 Å along its longest extension [25].

The system was subjected to an energy minimization and was then equilibrated for 1 ns at a temperature of 300 K and constant pressure of 0.1 MPa by applying a Langevin thermostat and the Langevin piston algorithm [31,32].

After the system had been prepared, diffusion simulations of a 3-ns duration in an NPT ensemble were started; for each system, we performed 20 simulations with varied initial cisplatin orientation. We used here conventional molecular dynamics (cMD) simulation, while in a previous publication [25], the technique of accelerated molecular dynamics (aMD) simulation [33,34] was employed. We used the software NAMD 2.10 [35] with the CHARMM27 force field [36]. VMD 1.9.3 [37] and Tachyon [38] were used to render the adsorption snapshots.

## 3. Results and Discussion

**Figure 1:**
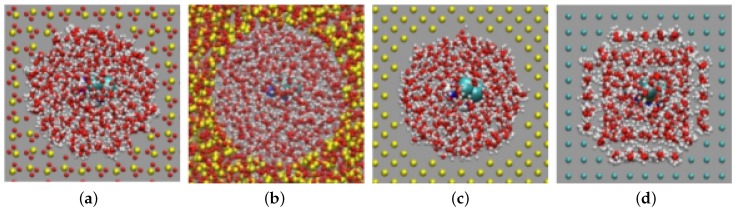
Cross-sectional view of the nanopore at the end of the equilibration stage showing the water hydration shells surrounding the pore walls. (**a**) Axial cristobalite pore; (**b**) amorphous silica; (**c**) silicon; (**d**) gold. Oxygen is colored red and hydrogen white.

In the course of the 1-ns equilibration process, the hydration shell of the pore walls strongly changed, and a hydration layer was established; see Figure 1. For Au, the effect was strongest, and a highly-ordered water shell was established around the Au wall with the hydrogen atoms oriented towards the wall. Indeed, it is known from ab initio calculations of the interaction of water molecules with Au surfaces that molecular configurations with the H atoms facing the wall are preferred [39]. In the ensuing diffusion simulations, we observed that cisplatin never penetrated this hydration layer; as a consequence, the adsorption bonding strength on Au was reduced as compared to a water-free Au surface. For the other surfaces, the amount of water ordering was smaller than for Au, since their interaction with water was less attractive.

In the following, we present and discuss the results of our simulations. The diffusion coefficients of cisplatin in the porous materials investigated are determined. Finally, we focus on the adsorption of the drug molecule during the simulation to the pore wall and investigate the main factors responsible for the adsorption process.

### 3.1. Diffusivity of Cisplatin in Inorganic Nanopores

The diffusivity of cisplatin in the pore can be determined from the one-dimensional version of the Einstein relation,

(1) $$\langle z^{2}\left(t\right)\rangle =2Dt+c,$$

where the constant offset *c* is used for an improvement of the large-time fit [40]. The left-hand part of this expression is known as the mean squared displacement (MSD) of the molecule. The coordinate *z* measures the motion along the pore axis. The average 〈⋯〉 is over the 20 trajectories simulated [40,41]. Equation (1) is valid at times that are long compared to the time scale at which the velocity autocorrelation vanishes; this is of the order of 1 ps. In our case, the molecule performed a trajectory of “hindered” diffusion, where each molecule went through phases of free diffusion inside the pore and through phases of adsorption on the pore wall. If the ensemble average in Equation (1) is performed over a sufficiently large number of these phases, the diffusive behavior can be calculated also in these cases of “hindered” diffusion. As our results below show, our systems indeed satisfied (more or less) this condition, as verified in the energy evolutions in Figure 6a, etc., below.

**Figure 2:**
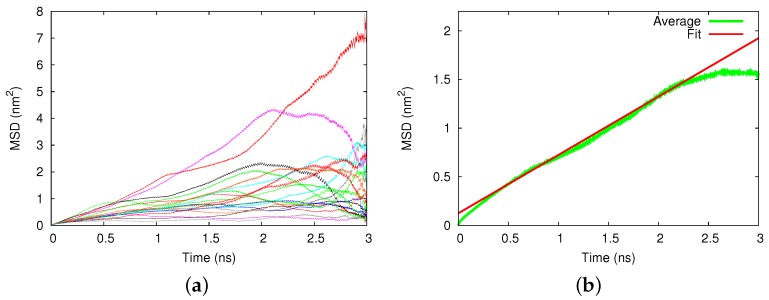
Diffusion of cisplatin in the gold nanopore. (**a**) Mean squared displacement (MSD) of 20 individual 3-ns diffusion runs. (**b**) Average over the individual runs, compared to a fit line, Equation (1).

Figure 2a displays an example of the square displacements of the 20 individual trajectories in a Au nanopore. Large deviations between the individual runs showed up. After averaging, Figure 2b, a diffusive time dependence, MSD ∝t, in agreement with Equation (1) showed up from which we determined the diffusion coefficient of D=150±22μm${}^{2}$/s. The error was estimated from the uncertainty of the fit of the slope *D* in Equation (1). To put this into perspective, we noted that the diffusion coefficient of cisplatin in pure water amounted to D=1990±90 μm${}^{2}$/s.

**Figure 3:**
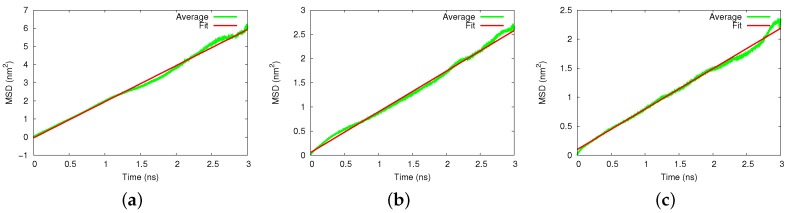
Diffusion of cisplatin in (**a**) silicon, (**b**) amorphous silica, and (**c**) the axial cristobalite pore. MSD, averaged over 20 individual 3-ns diffusion runs, are compared to a fit line, Equation (1).

We display in Figure 3 the averaged MSDs for diffusion in the silicon and amorphous silica pores. Again, the MSD data were averaged over 20 trajectories and allowed determining the pertinent diffusion coefficients. For the silicon pore, Figure 3a, we obtained a value of D=995±11 μm${}^{2}$/s. For the amorphous silica, we obtained D=420±45 μm${}^{2}$/s, for cristobalite (axial) D=348±35 μm${}^{2}$/s and for cristobalite (transverse) D=410±25 μm${}^{2}$/s.

**Figure 4:**
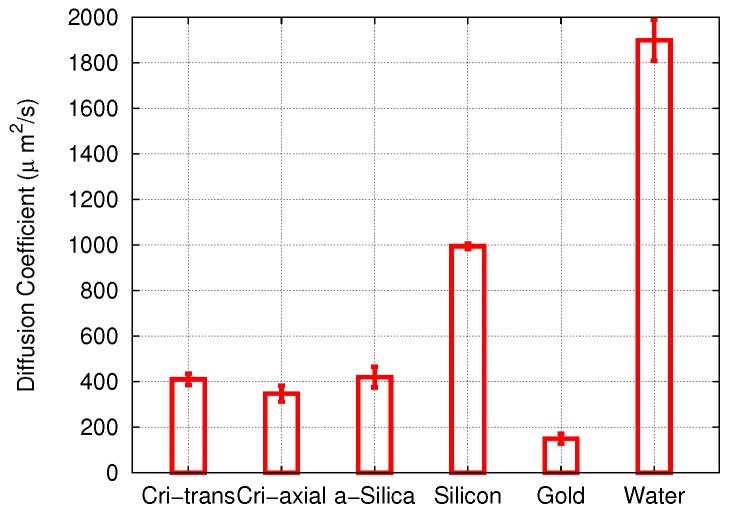
Diffusion coefficients of cisplatin (cis) in different pore materials and in water. Error bars were obtained from the uncertainty of the fit of the slope *D* in Equation (1).

These data were assembled in Figure 4 and compared to the diffusivity of cisplatin in bulk water. In all cases, the diffusion coefficient was smaller than in water, indicating that adsorption at the pore walls reduced the diffusion coefficient. The results for cisplatin diffusion in the three forms of SiO${}_{2}$ studied—amorphous silica and two different pores in cristobalite—were quite similar. Diffusion in the gold nanopore was most strongly affected, since there, the adsorption energy was highest. In the following, we shall examine the adsorption processes in the various pores in detail.

### 3.2. Adsorption at the Pore Walls

**Figure 5:**
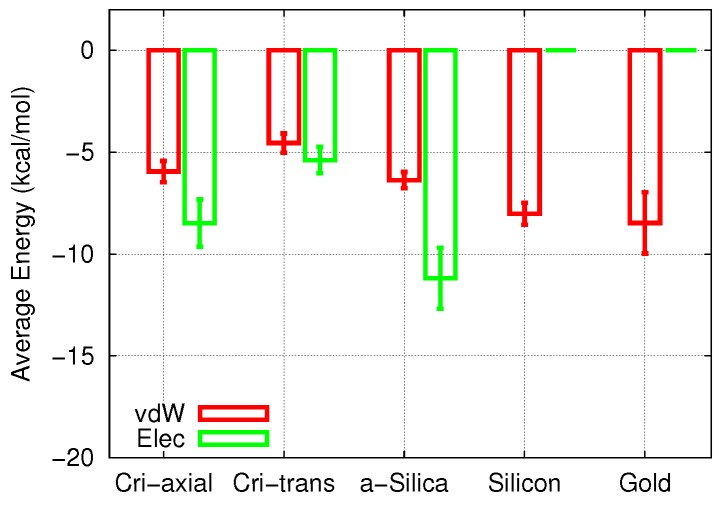
Average electrostatic (Elec) and vdW energies between the cisplatin molecule and the pore surface during the 3-ns diffusion process as a function of pore material. Error bars were obtained from the fluctuations of the energies along the trajectories.

In order to understand in more detail the reduced diffusion coefficients in the pores, we investigated the molecule adsorption at the pore walls. The interaction of cisplatin with the walls can most easily be quantified by the adsorption energy. Since cisplatin makes no bonds with the surfaces, we need only discuss electrostatic and vdW contributions. Figure 5 displays the adsorption energies, averaged over the 20 individual runs and the entire time period of the simulations.

In both the crystalline and the amorphous silica, the electrostatic energies slightly surpassed the vdW energies. Note that the electrostatic adsorption energies in the three SiO${}_{2}$ pores studied here were slightly different, while the vdW energies were more comparable. The vdW contribution in Si was comparable in size to that of the polar materials, while in Au, the vdW interaction was the strongest of all cases studied.

An interesting issue concerns the different effects that electrostatic and vdW interaction energies exert on the adsorption and the diffusion process. Si had a vdW energy that was comparable to silica; however, the additional attraction provided by the electrostatic forces made the total adsorption energy of cisplatin on silica larger than on silicon. As a consequence, the diffusion in the silica pores was more strongly hindered than in silicon.

In order to assess the implications of the adsorption on cisplatin diffusion in the pore, we will now discuss the time evolution of the adsorption energies for representative trajectories for the materials studied here.

**Figure 6:**
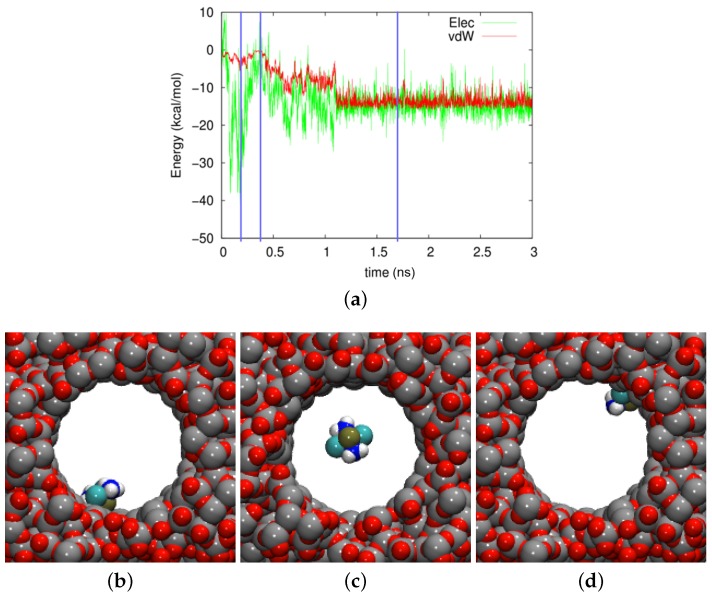
(**a**) Evolution of the electrostatic (Elec) and vdW energies between the cisplatin molecule and the amorphous silica pore during the diffusion process. (**b**–**d**) show snapshots at times of (**b**) 182 ps, (**c**) 388 ps, and (**d**) 1700 ps. These times are marked in (**a**) by vertical blue lines.

In Figure 6, the time evolution of the adsorption energy is shown in the amorphous silica pore. The trajectory was characterized by strong fluctuations, both in the vdW and the electrostatic part. The vdW contribution was always attractive, its size changing with the distance of the molecule to the wall. However, the electrostatic part exhibited both positive and negative signs and can hence be both repulsive and attractive; this was mainly controlled by the orientation of the molecule, which determined the atoms facing the wall. As Figure 5 showed, the averaged electrostatic energy was attractive, −11.18 kcal/mol.

Three snapshots are shown in Figure 6b–d. In Figure 6c, the cisplatin molecule is almost in the middle of the pore. We considered the molecule as desorbed in this case, the vdW energy amounting to −0.29 kcal/mol and the electrostatic energy to +0.18 kcal/mol. In contrast, Figure 6a shows a strongly-adsorbed molecule where the electrostatic energy was considerably increased to the amount −39.97 kcal/mol, and the vdW energy had almost the highest value in the trajectory, −5.10 kcal/mol. The case of Figure 6d shows a more weakly-adsorbed state, which, however, persisted for a longer period of time.

**Figure 7:**
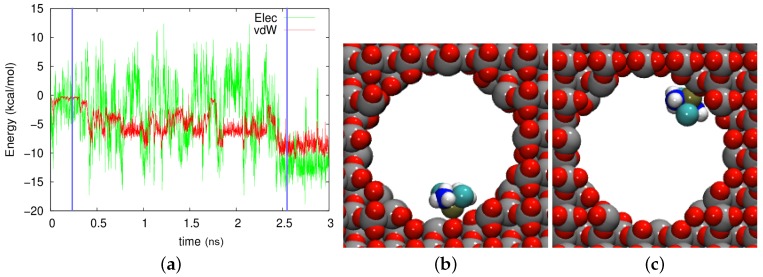
(**a**) Evolution of the electrostatic (Elec) and vdW energies between the cisplatin molecule and the axial cristobalite pore during the diffusion process. (**b**,**c**) show snapshots at times of (**b**) 433 ps and (**c**) 2572 ps. These times are marked in (**a**) by vertical blue lines.

In the axial pore in crystalline cristobalite, Figure 7, even more fluctuations and more varied adsorption states can be identified. Two of them (with the Pt atom facing the wall) are displayed in Figure 7b,c. While the adsorption geometry looked similar in both cases, the energies were quite different: −8.74 kcal/mol electrostatic and −8.99 kcal/mol vdW energies in Figure 7b and −13.68 kcal/mol electrostatic and −8.57 kcal/mol vdW energies in Figure 7c. This difference stems from the fact that the Pt atom was close to a row of oxygen atoms at 433 ps, but it was close to row of Si atoms at 2572 ps. In this trajectory as well, large fluctuations in the electrostatic field dominated at all times.

We conclude that in the polar pores, the cisplatin motion was governed by short-lasting adsorption and desorption events. High fluctuations of the electrostatic energy were caused by the strong orientation dependence of the electrostatic interaction energy and were the reason that adsorption never occurred on longer time scales. These results did not change qualitatively if the polar wall material was crystalline. As a result, the cisplatin diffusion coefficient was comparatively large, even though the adsorption episodes reduced it as compared to the case of pure water.

**Figure 8:**
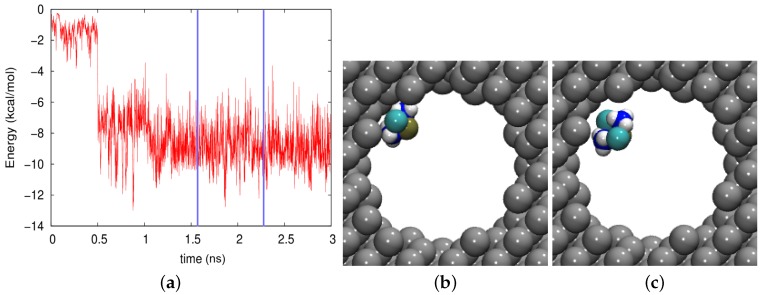
(**a**) Evolution of the vdW energy between the cisplatin molecule and the silicon pore during the diffusion process. (**b**,**c**) show snapshots at times of (**b**) 1584 ps and (**c**) 2325 ps. These times are marked in (**a**) by vertical blue lines.

In silicon, where no electrostatic effects are present, the adsorption is entirely governed by the vdW interaction, Figure 8a. Episodes where the molecule was in the middle of the pore and the interaction energy was small were rare. Often, however, the molecule was adsorbed at the wall and felt a high vdW energy; an example is given in Figure 8b, where the vdW energy amounted to −10.16 kcal/mol. An intermediate case, with a different cisplatin orientation, is shown in Figure 8c. The cisplatin trajectory can hence be described as a process of “hindered diffusion”, where the free motion along the pore axis was interrupted by episodes in which the molecule was stuck to the wall. As a consequence, the diffusion coefficient was considerably decreased with respect to that in bulk water.

**Figure 9:**
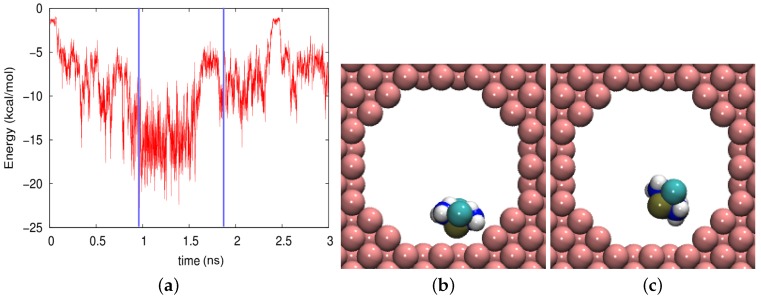
(**a**) Evolution of the vdW energy between the cisplatin molecule and the gold pore during the diffusion process. (**b**,**c**) show snapshots at times of (**b**) 968 ps and (**c**) 1884 ps. These times are marked in (**a**) by vertical blue lines.

The case of the Au nanopore was even more extreme, since the vdW forces here were higher. Figure 9a demonstrates that within less than 100 ps, the molecule had come close to the wall, leading to an attractive vdW interaction. During most of its trajectory, the molecule would not escape the wall; only at around 2.5 ns, it came free for a while. However, depending on the orientation of the molecule at the wall, the adsorption energy may considerably change. The lowest adsorption energy of around −23 kcal/mol was obtained if the cisplatin molecule was oriented with the Pt atom facing the wall, Figure 9b; in other episodes, it changed its orientation to H atoms facing the wall, where it had only −6 kcal/mol adsorption energy, Figure 9c.

We conclude that the motion of cisplatin in a nonpolar pore was hindered by adsorption events. In cases of small vdW energy, desorption led to episodes of free motion along the channel axis and correspondingly to only a moderate reduction of the diffusion coefficient as compared to that in bulk water. In cases of large vdW energies, cisplatin was adsorbed at the wall for longer episodes, leading to a further reduction of the diffusion coefficient.

We note that in a previous paper [25], we used accelerated molecular dynamics [33,34] in order to sample the molecular phase space for adsorption more efficiently. While this is true, on the other side, acceleration affected the free diffusive motion of the drug molecule, such that we believe that our present results on diffusion as obtained by conventional (i.e., non-accelerated) MD were better justified. In addition, we used a longer equilibration time for preparing the system (1 ns), which allowed the system to establish a hydration layer on top of the wall surfaces. This layer reduced the adsorption energies considerably, in particular for the case of the Au nanopore. In an unequilibrated system, cisplatin adsorbed with −35.74 kcal/mol; in our equilibrated simulation, it was only −8.74 kcal/mol.

## 4. Conclusions

Adsorption on the wall affected diffusion in the 10-nm pores, and unsurprisingly, the higher the adsorption energy, the smaller the diffusion coefficient. In nonpolar materials, drug diffusion was hindered by adsorption events. In cases of small vdW energy, desorption led to episodes of free motion along the channel axis and correspondingly to only a moderate reduction of the diffusion coefficient as compared to that in bulk water. In cases of large vdW energies, cisplatin was adsorbed for longer episodes at the wall, leading to a stronger reduction of the diffusion coefficient.

In polar materials, on the other hand, the cisplatin motion was governed by short-lasting adsorption and desorption events. High fluctuations of the electrostatic energy were caused by the strong orientation dependence of the electrostatic interaction energy and prevented long-lasting adsorption.

We found the behavior of amorphous silica to follow that of crystalline silica closely. We concluded that both crystalline and amorphous nanoporous silica could well be used as a container for cisplatin drug delivery, in particular if pore diameters larger than 2 nm were used.
